# Supplementary material for: A Critical View of the Application of the APEX Software (Aqueous Photochemistry of Environmentally-Occurring Xenobiotics) to Predict Photoreaction Kinetics in Surface Freshwaters
Source: Molecules. 2019 Dec 18;25(1):9. doi: 10.3390/molecules25010009 (PMC7017383; doi:10.3390/molecules25010009)
Supplement: Supplementary file 1 [file molecules-25-00009-s001.zip › 3.2.4_gcc-4.4.0.zip!/3.2.4_gcc-4.4.0/3.2.4_gcc-4.4.0/doc/octave/html/interpreter/Sparse-Matrices-with-Mex_002dFiles.html]

Sparse Matrices with Mex-Files - Untitled


Next: Calling Other Functions in Mex-Files,
Previous: Structures with Mex-Files,
Up: Mex-Files


---

#### A.2.6 Sparse Matrices with Mex-Files

The Octave format for sparse matrices is identical to the mex format in
that it is a compressed column sparse format. Also in both, sparse
matrices are required to be two-dimensional. The only difference is that
the real and imaginary parts of the matrix are stored separately.

The mex-file interface, as well as using `mxGetM`, `mxGetN`,
`mxSetM`, `mxSetN`, `mxGetPr`, `mxGetPi`,
`mxSetPr` and `mxSetPi`, the mex-file interface supplies the
functions

```
     mwIndex *mxGetIr (const mxArray *ptr);
     mwIndex *mxGetJc (const mxArray *ptr);
     mwSize mxGetNzmax (const mxArray *ptr);
     
     void mxSetIr (mxArray *ptr, mwIndex *ir);
     void mxSetJc (mxArray *ptr, mwIndex *jc);
     void mxSetNzmax (mxArray *ptr, mwSize nzmax);
```

`mxGetNzmax` gets the maximum number of elements that can be stored
in the sparse matrix. This is not necessarily the number of non-zero
elements in the sparse matrix. `mxGetJc` returns an array with one
additional value than the number of columns in the sparse matrix. The
difference between consecutive values of the array returned by
`mxGetJc` define the number of non-zero elements in each column of
the sparse matrix. Therefore

```
     mwSize nz, n;
     mwIndex *Jc;
     mxArray *m;
     ...
     n = mxGetN (m);
     Jc = mxGetJc (m);
     nz = Jc[n];
```

returns the actual number of non-zero elements stored in the matrix in
`nz`. As the arrays returned by `mxGetPr` and `mxGetPi`
only contain the non-zero values of the matrix, we also need a pointer
to the rows of the non-zero elements, and this is given by
`mxGetIr`. A complete example of the use of sparse matrices in
mex-files is given by the file mysparse.c as seen below

```
```
     #include "mex.h"
     
     void
     mexFunction (int nlhs, mxArray *plhs[], int nrhs, 
     	     const mxArray *prhs[])
     {
       mwSize n, m, nz;
       mxArray *v;
       mwIndex i;
       double *pr, *pi;
       double *pr2, *pi2;
       mwIndex *ir, *jc;
       mwIndex *ir2, *jc2;
       
       if (nrhs != 1 || ! mxIsSparse (prhs[0]))
         mexErrMsgTxt ("expects sparse matrix");
     
       m = mxGetM (prhs [0]);
       n = mxGetN (prhs [0]);
       nz = mxGetNzmax (prhs [0]);
       
       if (mxIsComplex (prhs[0]))
         {
           mexPrintf ("Matrix is %d-by-%d complex",
     		 " sparse matrix", m, n);
           mexPrintf (" with %d elements\n", nz);
     
           pr = mxGetPr (prhs[0]);
           pi = mxGetPi (prhs[0]);
           ir = mxGetIr (prhs[0]);
           jc = mxGetJc (prhs[0]);
     
           i = n;
           while (jc[i] == jc[i-1] && i != 0) i--;
           mexPrintf ("last non-zero element (%d, %d) =", 
     		 ir[nz-1]+ 1, i);
           mexPrintf (" (%g, %g)\n", pr[nz-1], pi[nz-1]);
     
           v = mxCreateSparse (m, n, nz, mxCOMPLEX);
           pr2 = mxGetPr (v);
           pi2 = mxGetPi (v);
           ir2 = mxGetIr (v);
           jc2 = mxGetJc (v);
           
           for (i = 0; i < nz; i++)
             {
               pr2[i] = 2 * pr[i];
               pi2[i] = 2 * pi[i];
               ir2[i] = ir[i];
             }
           for (i = 0; i < n + 1; i++)
             jc2[i] = jc[i];
     
           if (nlhs > 0)
             plhs[0] = v;
         }
       else if (mxIsLogical (prhs[0]))
         {
           bool *pbr, *pbr2;
           mexPrintf ("Matrix is %d-by-%d logical",
     		 " sparse matrix", m, n);
           mexPrintf (" with %d elements\n", nz);
     
           pbr = mxGetLogicals (prhs[0]);
           ir = mxGetIr (prhs[0]);
           jc = mxGetJc (prhs[0]);
     
           i = n;
           while (jc[i] == jc[i-1] && i != 0) i--;
           mexPrintf ("last non-zero element (%d, %d) = %d\n",
                      ir[nz-1]+ 1, i, pbr[nz-1]);
     
           v = mxCreateSparseLogicalMatrix (m, n, nz);
           pbr2 = mxGetLogicals (v);
           ir2 = mxGetIr (v);
           jc2 = mxGetJc (v);
           
           for (i = 0; i < nz; i++)
             {
               pbr2[i] = pbr[i];
               ir2[i] = ir[i];
             }
           for (i = 0; i < n + 1; i++)
             jc2[i] = jc[i];
     
           if (nlhs > 0)
             plhs[0] = v;
         }
       else
         {
           mexPrintf ("Matrix is %d-by-%d real",
     		 " sparse matrix", m, n);
           mexPrintf (" with %d elements\n", nz);
     
           pr = mxGetPr (prhs[0]);
           ir = mxGetIr (prhs[0]);
           jc = mxGetJc (prhs[0]);
     
           i = n;
           while (jc[i] == jc[i-1] && i != 0) i--;
           mexPrintf ("last non-zero element (%d, %d) = %g\n",
                     ir[nz-1]+ 1, i, pr[nz-1]);
     
           v = mxCreateSparse (m, n, nz, mxREAL);
           pr2 = mxGetPr (v);
           ir2 = mxGetIr (v);
           jc2 = mxGetJc (v);
           
           for (i = 0; i < nz; i++)
             {
               pr2[i] = 2 * pr[i];
               ir2[i] = ir[i];
             }
           for (i = 0; i < n + 1; i++)
             jc2[i] = jc[i];
     
           if (nlhs > 0)
             plhs[0] = v;
         }
     }
```
```
